# Multimodal imaging in a case of a congenital retinal macrovessel associated with a retinal cavernous hemangioma: a case report

**DOI:** 10.1186/s12886-020-1326-4

**Published:** 2020-02-04

**Authors:** Walid Zbiba, Issam Eddine Elleuch, Sana Sayadi, Meriem Laadheri

**Affiliations:** Ophthalmology department, Mohamed Taher Maamouri hospital, Faculty of Medicine Tunis El Manar, 03 Rue Abou Zid El Hilali, 2010 Manouba, Tunisia

**Keywords:** Cavernous hemangioma, Congenital macrovessel, Chorioretinal folds, Optical coherence tomography angiography

## Abstract

**Background:**

To report the results of multimodal imaging in a case of a congenital retinal macrovessel associated with a retinal cavernous hemangioma.

**Case presentation:**

A 52-year-old female patient presented with progressive vision loss in the right eye. BCVA was 8/20 in the right eye and 18/20 in the left eye. Fundus examination of the right eye showed an aberrant retinal macrovessel arising from the inferior temporal major vein. It crossed the foveal area and overstepped to the superior retina. A “brunch of grapes” shaped retinal lesions arised from the macrovessel. Fluorescein angiography showed saccular lesions that filled slowly during the venous phase and became brightly hyperfluorescent saccular caps. SS-OCT of the right eye revealed a highly back-scattering hyper-reflective vessel across the fovea with shadow effect and adhesions between the vitreous and the aberrant macrovessel. It also revealed hypo reflective saccules with hyperreflective borders located in the inner retina corresponding to the cavernous retinal hemangioma. Retinal pigment epithelium undulations and vascular dilations at the level of Haller’s layer were observed in both eyes. Indocyanine green angiography revealed chroidal vascular dilatations in both eyes in the late phase. OCT-A showed the aberrant vessel emerging from the inferior temporal vein and splitting the foveal avascular zone horizontally. RCH appeared as small heterogeneous saccular flow areas associated with focal capillary hypo perfusion areas. Asymmetry and distorsion of the foveal avascular zone were also noticed.

A diagnosis of retinal macrovessel associated with a retinal cavernous hemangioma was made.

**Conclusions:**

Congenital retinal macrovessels and retinal cavernous hemangioma are benign lesions. Their association is rare. Abnormal vascular development is likely to be responsible for their association. Swept source OCT and OCT angiography may be of a great contribution to better evaluate these retinal vascular disorders.

## Background

Congenital retinal macrovessels (CRMs) are aberrant vessels typically veins, crossing the horizontal raphe in the macular region that were first described in 1869 [1]. CRMs are usually asymptomatic. They may be observed in association with other vascular abnormalities and be responsible for vision loss [1].

Herein, we report the results of multimodal imaging of a CRM associated with retinal vascular abnormalities consistent with cavernous hemangioma.

To our knowledge, this is the first report of this association imaged by optical coherence tomography angiography (OCTA) in the indexed peer-reviewed literature.

## Case presentation

**Fig. 1:**
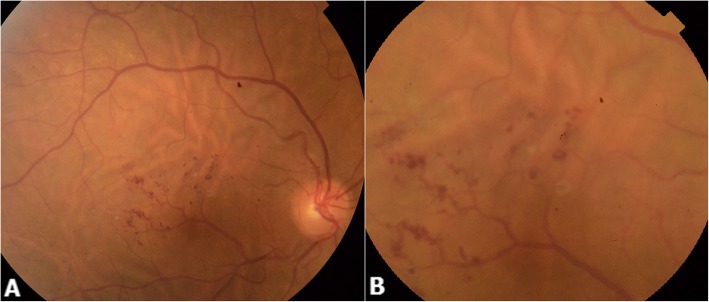
Color fundus photography of the right eye (**a** and **b**) showing an aberrant retinal macrovessel arising from the inferior temporal major vein, crossing the foveal area, and oversteppping to the superior retina (**a**). A two optic disc diameter sized “brunch of grapes” shaped retinal lesions arise from the aberrant macrovessel (**b**). Dilated choroidal vessels with choroidal folds also are visible. (**a** and **b**)

**Fig. 2:**
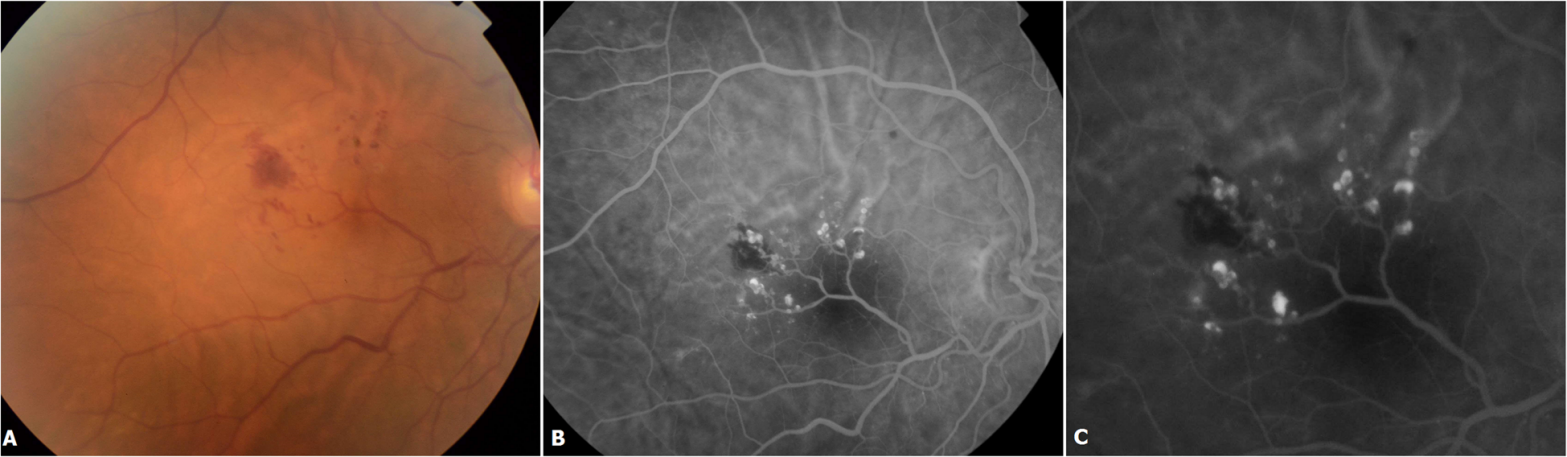
Color fundus photography showing an intra-retinal hemorrhage located temporally to the fovea (**a**). Fluorescein angiography showing saccular lesions that fill slowly during the venous phase (**b**) and become brightly hyperfluorescent saccular caps with no leakage (**c**). The aberrant vessel’s filling is normal (**b**)

A 52-year-old female patient first presented to our department with progressive painless vision loss in the right eye. She had no history of trauma or ocular surgery and had a family history of an open angle glaucoma. On examination she was moderately hypermetropic in both eyes (RE: + 3.5 Dp; LE: + 2 Dp). BCVA was 8/20 in the right eye and 18/20 in the left eye. Anterior segment examination and intraocular pressure in both eyes were normal. Fundus examination of the right eye showed an aberrant retinal macrovessel arising from the inferior temporal major vein. It crossed the foveal area and overstepped to the superior retina. A two optic disc diameter sized “brunch of grapes” shaped retinal lesions arised from the aberrant macrovessel (Fig. 1). Fluorescein angiography showed saccular lesions that filled slowly during the venous phase and became brightly hyperfluorescent saccular caps, which were consistent with a diagnosis of retinal cavernous hemangioma. No leaking was noticed. The aberrant vessel’s filling was normal and there were no specific alterations (Fig. 2).

**Fig. 3:**
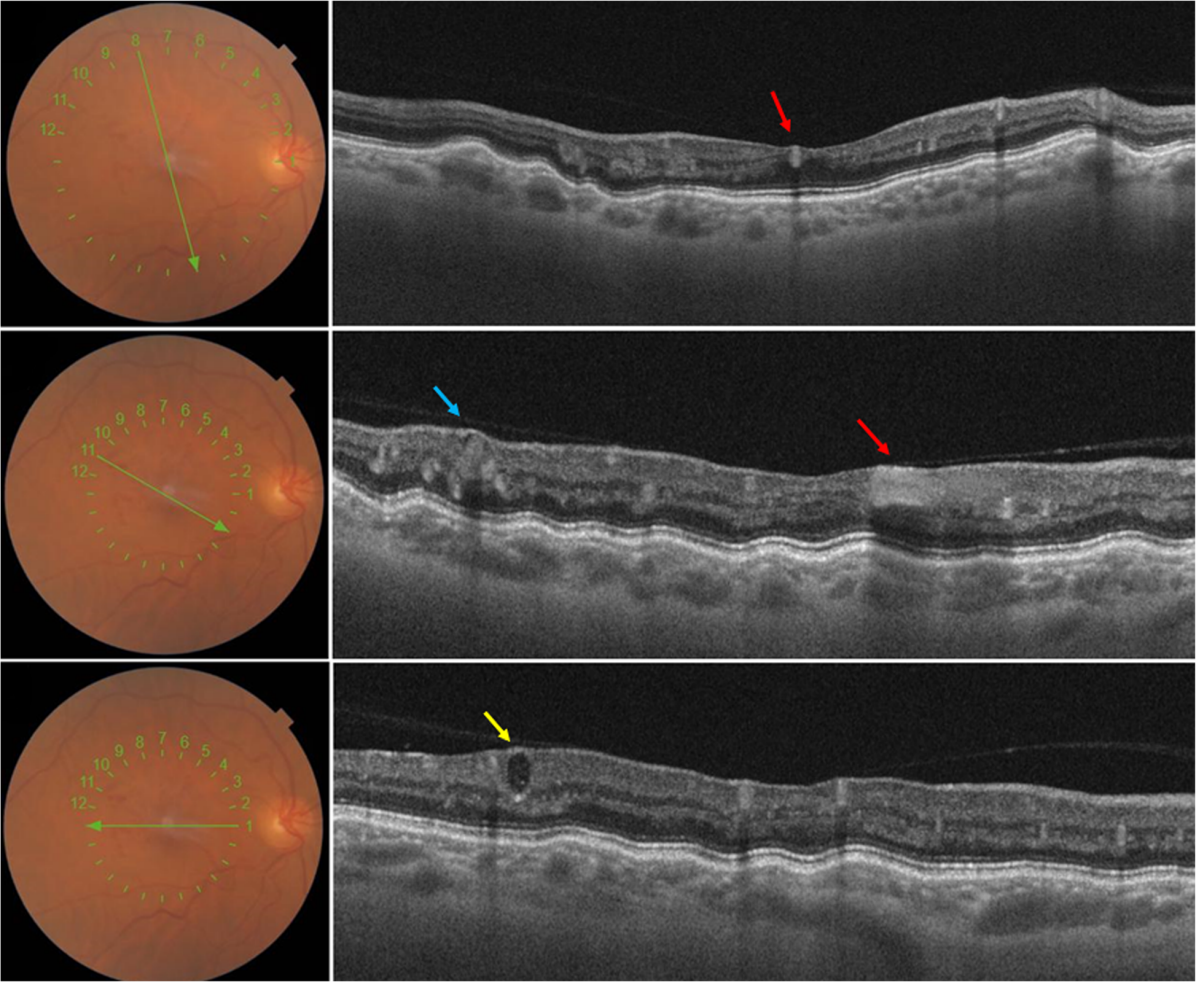
Optical coherence tomography (B scan) of the right eye showing highly back-scattering hyper-reflective vessel crossing the fovea (red arrow) with shadow effect (B) and adhesions between the posterior hyaloid and the aberrant macrovessel (B). Hypo reflective saccules with hyper reflective borders located in the inner retina corresponding to the cavernous retinal hemangioma (CRH) (blue arrow). Variable degrees of hypo and hyper reflectivities were observed inside these cystic lesions (Yellow arrow)

SS-OCT analysis of the right eye revealed a highly back-scattering hyper-reflective vessel across the fovea with shadow effect (Fig. 3a) and adhesions between the vitreous and the aberrant macrovessel (Fig. 3b). The highly scattering structure of the RPE was responsible for a weak but identifiable signal. The retinal structures between the RPE and the vessel wall were not visible due to their low scattering properties (Fig. 3c).

Central retina was thinner in the right eye compared to the left one. It also revealed hypo reflective saccules with hyperreflective borders located in the internal retinal layers corresponding to the cavernous retinal hemangioma (CRH). Variable degrees of hypo and hyper reflectivity were observed inside these cystic lesions (Fig. 3b, c).

**Fig. 4:**
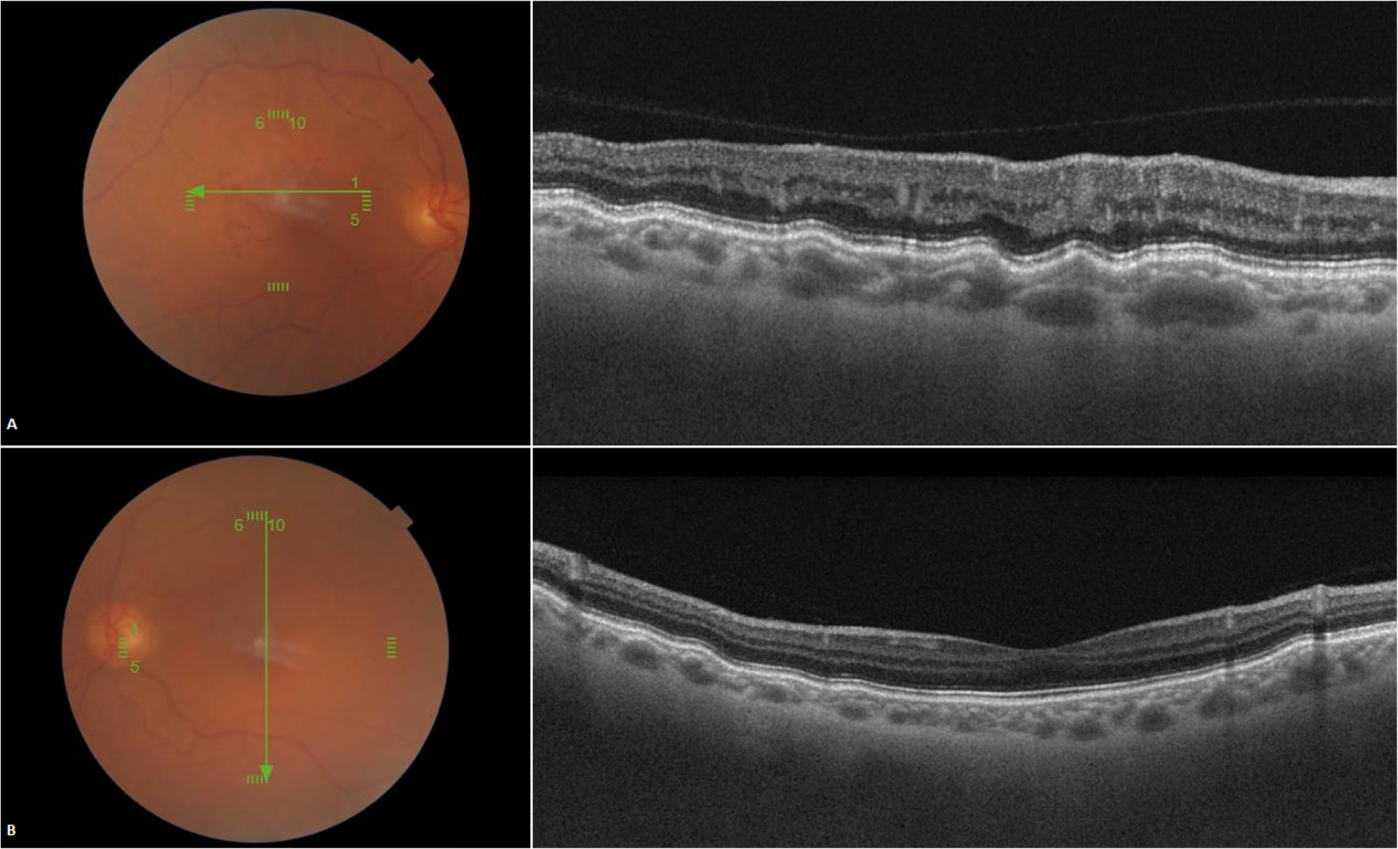
Optical coherence tomography (B scan) showing retinal pigment epithelium undulations in both eyes (**a**, **b**) more pronounced in the right eye (**a**), choroidal thickness is within normal limits, and there are vascular dilations at the level of Haller’s layer in both eyes more marked in the right eye

**Fig. 5:**
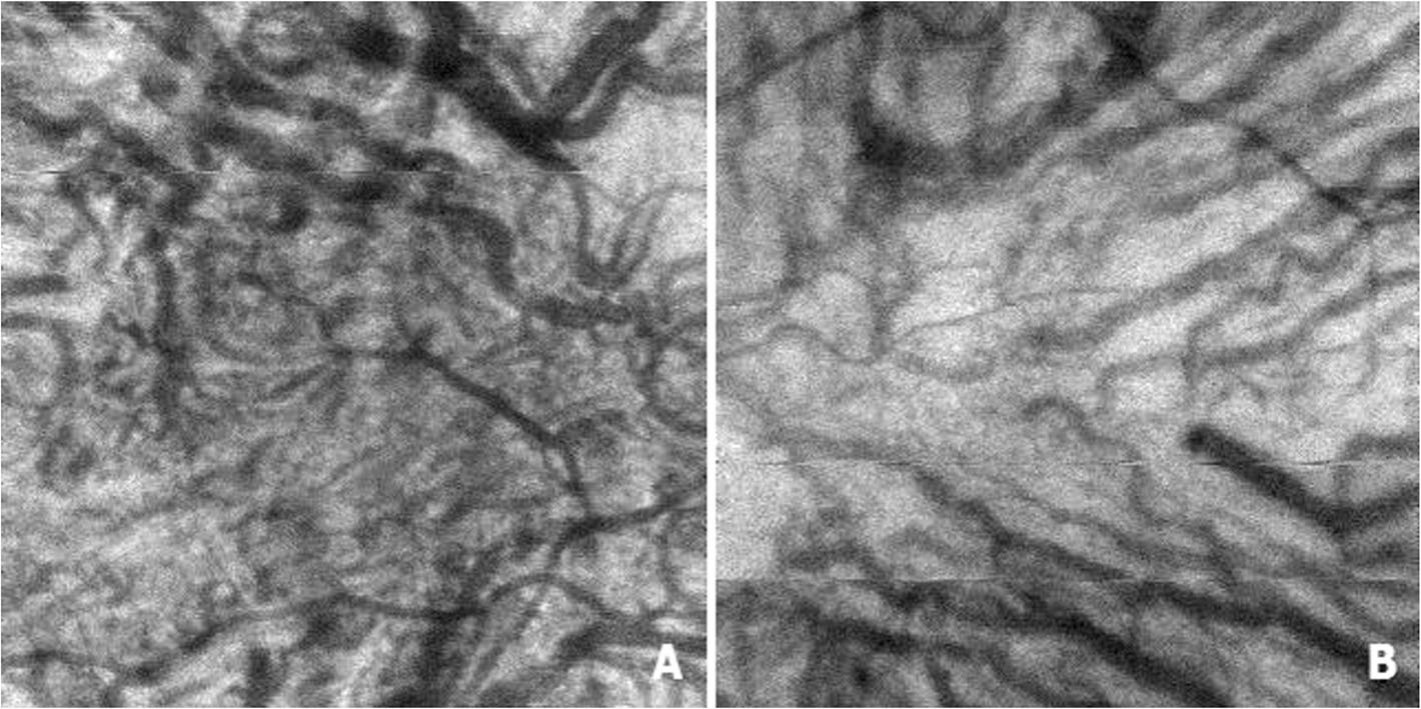
En face OCT of the right eye (**a**), left eye (**b**) showing bilateral choroidal vascular dilatations

There was no evidence of retinal layers loss, macular edema or retinal detachment and foveal profile was preserved. Retinal pigment epithelium undulations were observed in both eyes and were more pronounced in the affected eye, choroidal thickness was within normal limits but there were vascular dilations at the level of Haller’s layer in both eyes more pronounced in the right eye (Fig. 4). En face OCT showed choroidal vascular dilations in both eyes (Fig. 5).

**Fig. 6:**
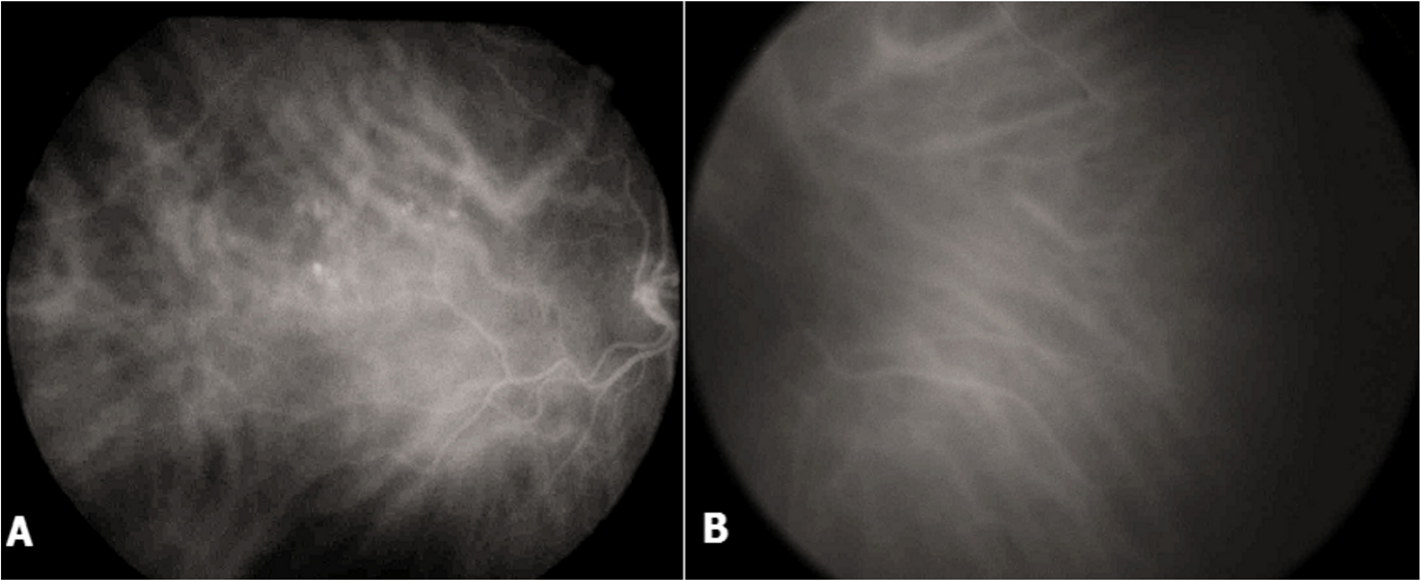
Indocyanine green angiography of the right eye (**a**), left eye (**b**) showing bilateral choroidal vascular dilatations more marked in the right eye. Aneurysmal lesions are present in the right eye (**a**)

Indocyanine green angiography revealed choroidal vascular dilatations in both eyes in the late phase, these abnormalities were more marked in the left eye (Fig. 6).

**Fig. 7:**
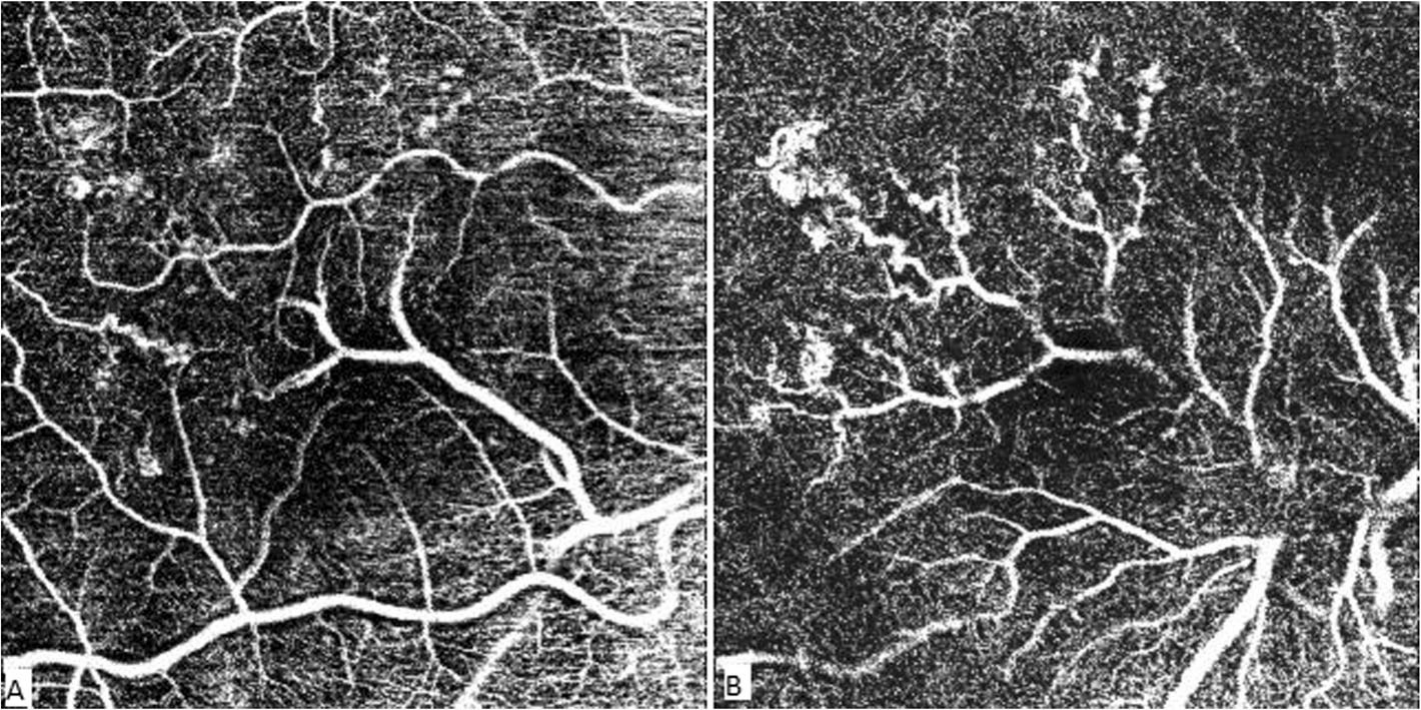
OCT-A of the superficial plexus (SCP) (**a**) showing the aberrant vessel emerging from the inferior temporal vein and branching at the level of the fovea. It splits the foveal avascular zone (FAZ) horizontally and causes an asymmetry and distortion of the FAZ (**a**). The terminal branches of the aberrant vessels are observed at the level of the deep capillary plexus (DCP) (**b**). Retinal cavernous hemangiomas (RCH) appear as small heterogeneous saccular flow areas identified on the deep plexus along the branches of the macrovessel (**b**). Hypo perfusion areas surrounding the vascular anomalies are visible in both the SCP and DCP (**a** and **b**)

OCT-A showed in the right eye the aberrant vessel emerging from the inferior temporal vein and splitting the foveal avascular zone horizontally into two parts. RCH appeared as small heterogeneous saccular flow areas associated with focal capillary hypo perfusion areas. Asymmetry and distorsion of the foveal avascular zone were also noticed (Fig. 7).

A diagnosis of retinal macrovessel associated with a retinal cavernous hemangioma was made based on clinical and conventional angiographic findings. General examination disclosed no abnormal neurological signs or cutaneous hemangiomas. Cerebral MRI and abdominal ultrasonography ruled out the presence of associated cerebral and abdominal vascular abnormalities.

During a three-year follow up period, the patient developed an intra-retinal hemorrhage located temporally to the fovea that resolved spontaneously in the affected eye. Clinical and OCT findings remained unchanged. We noted a progressive bilateral vision loss related to a senile bilateral nuclear cataract, visual acuity at the last follow-up examination was 6/20 in both eyes.

## Discussion and conclusions

Congenital retinal macrovessels (CRMs) are aberrant vessels, crossing the horizontal raphe in the macular region and having large tributaries, they are predominantly retinal veins [1, 2] originating from the temporal arcade, but could also originate from a retinal artery in 25% of cases or may originate from both arteries and veins.

When isolated, CRMs are usually benign and non-vision threatening and most often an incidental finding. However, in children they may lead to amblyopia [3] attributed to the macrovessel crossing the foveola or to its branches originating near the fovea which may alter its proper developement. In adults, decreased visual acuity is rare and can result from angioscotoma caused by decreased retinal sensitivity in the area crossed by the macrovessel, distortion of the foveal architecture or foveal ectopia [4]. In our case, the branches of the CRM originated at the fovea, which may have altered foveal development, and this could explain the decreased vision at presentation in the affected eye in our patient.

CRMs rarely occurred in conjunction with other retinal vascular pathologies including retinal cavernous hemangiomas [5], macroaneurysms [2] and telangiectasias [6]. In these cases, CRMs may as well be associated with complications including macular hemorrhage as seen in our case, tractional retinal detachment, macular ischemia, vitreous hemorrhage, macular exudation and branch retinal artery occlusion. Associated brain vascular anomalies like in Wyburn-Mason’s syndrome can be detected by neuroimaging examination. Systemic hemangiomas such as cerebral and abdominal ones may be associated with retinal ones, but none of these systemic lesions were found in our case.

FA in CRMs demonstrates five common findings including [7]: 1.early filling and delayed emptying of the macrovessel 2.tortuosity of the macrovessel with passage of one of the terminal branches across the macular area 3.presence of anastomoses between the aberrant vessels and the major retinal vessels 4.microvascular capillary bed anomalies such as arteriovenous anastomoses, capillary non perfusion and dilated surrounding capillary plexus 5.dye leakage from compromised microvascular bed or blocked fluorescence from the presence of preretinal hemorrhage. All these findings were seen in our patient.

OCT revealed shadowing behind the vessel, this demonstrates that the wall and the blood inside the aberrant vessel blood are highly scattering and therefore prevented most of the OCT beam from penetrating deeper layers. This may explain the angioscotomas seen by patients.

CRM was responsible for a distortion of the FAZ and for areas of capillary dropout mainly in the deep capillary plexus in the affected eye, thus, OCT-A may be of a great usefulness in evaluating microvascular changes associated with CRM crossing the foveal region.

Cavernous hemangioma is a rare benign retinal vascular tumor [8] in which pathologic vessels dilate and form cavities in which blood flow is slow or absent. It presents as a cluster of saccular, grape shaped aneuvrysms of dark red color located in the inner retina or at the surface of the optic disc, they usually occur at the mid periphery, and originates from normal retinal veins [9].

In our case, the hemangioma was located in the macular region and appeared along the course of the retinal macrovessel. This might suggest a common pahogenesis for these two conditions.

RCH may be associated with choroidal hemangioma, cutaneous and central nervous system angiomatous lesions.

FA typically shows oval hypofluorescent lesions that slowly fill with dye as the angiogram progresses, these lesions have fluorescence capping [10], which is secondary to erythrocyte sedimentation within the aneurysms, no extravascular leakage of dye is usually observed [11].

SS-OCT revealed multiple cystic formations with a hyperreflective boundries, which may indicate that the vesicules have a normal blood–retinal barrier. This correlates with the absence of leakage in the late phase of FA.

Different degrees of hyper reflectivity within these vesicules was observed and may be due to different plasma/erythrocyte sedimentation levels inside the aneurysms.

**Fig. 8:**
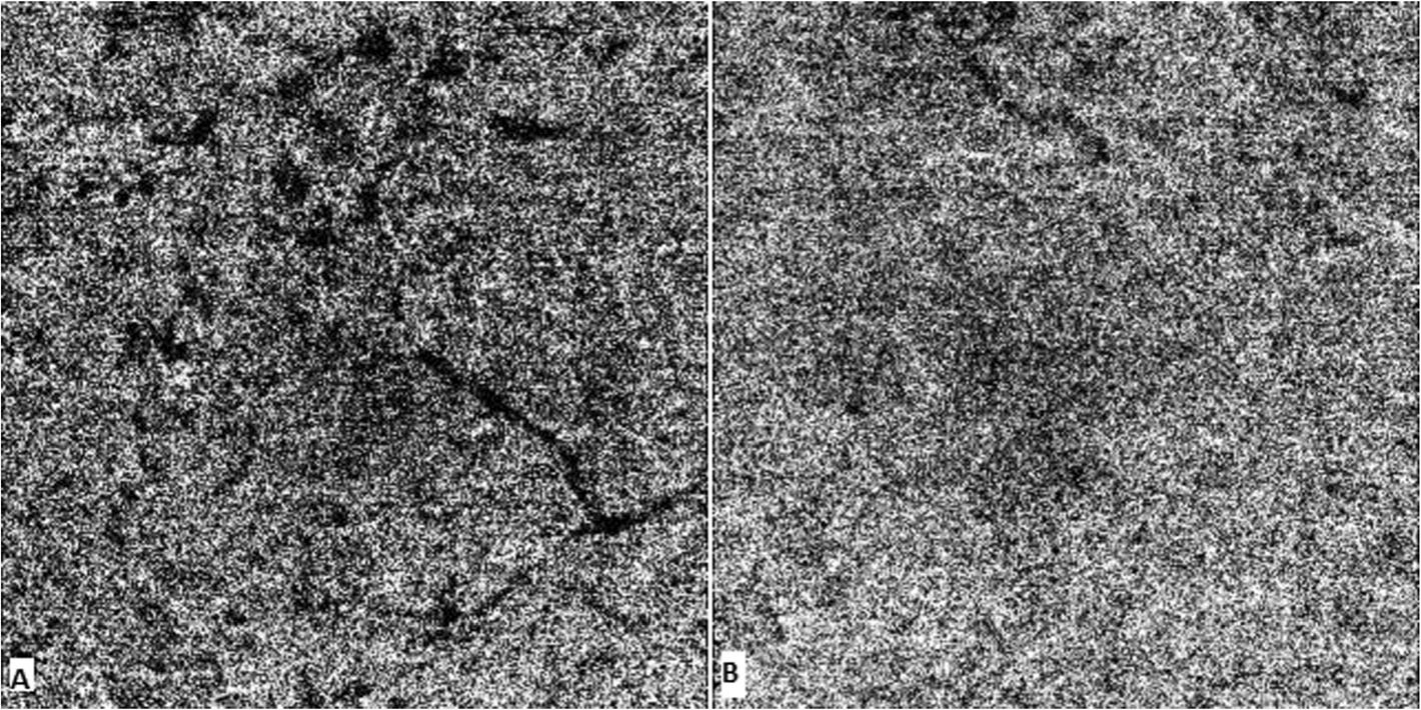
Right eye OCT-A image of choriocapillaris illustrates multifocal hypointense areas that precisely colocalize with the aberent vessel and the cavernous hemangiomas (**a**). Flow in the Choriocapillaris was normal in the left eye (**b**)

OCT-A detected flow within the tumor in both superficial and deep plexus that matched the saccular lesions seen in FA but was not able to detect flow in all of them. This was probably due to slow or absent flow within the hemangiomas, which correlates with different levels of erythrocyte sedimentation levels shown in SS-OCT. The tumors altered the surrounding microvascular architechture as shown by the presence of areas of capillary dropout surrounding the tumors that were not clearly identified in FA. OCT-A also showed alterations at the level of choriocapillaris, mainly caused by projection artifacts from the retinal lesions (Fig. 8).

The chorioretinal folds and corresponding RPE undulations on OCT observed in our case were probably caused by choroidal vessel dilation, demonstrated nicely by OCT and ICGA. To our knowledge, this is the first report showing an association between a retinal cavernous hemangioma, a congenital retinal macrovessel and bilateral choroidal vessels dilation. Our study may emphasize the role of abnormal vascular development as a common pathogenesis of these lesions.

Congenital retinal macrovessels and retinal cavernous hemangioma are benign lesions. Their association is rare. Abnormal vascular development is likely to be responsible for this presentation. Previously, CRMs and RCH have been examined using FA. Swept source OCT and OCT angiography may be of a great contribution to better evaluate these retinal vascular disorders.

## Data Availability

All data supporting our findings are contained within the manuscript.

## Abbreviations

BCVA: Best corrected visual acuity
CRMs: Congenital retinal macrovessels
Dp: Diopters
FA: Fluorescein angiography
ICGA: Indocyanine green angiography
LE: Left eye
OCTA: Optical coherence tomography angiography
RCH: Retinal cavernous hemangioma
RE: Right eye
RPE: Retinal pigment epithelium
SS-OCT: Swept-source optical coherence tomography

## References

[1] Strampe MR, Wirostko WJ, Carroll J (2017). A case of congenital retinal macrovessel in an otherwise normal eye. Am J Ophthalmol Case Rep.

[2] Goel N, Kumar V, Seth A, Ghosh B (2014). Branch retinal artery occlusion associated with congenital retinal macrovessel. Oman J Ophthalmol.

[3] Bhatia HK. Congenital retinal macrovessel with normal visual acuity: a case report. Int J Ophthalmolo Clin Res. 2015;2(2):017.

[4] Sanfilippo CJ, Sarraf D (2015). Congenital macrovessel associated with cystoid macular edema and an ipsilateral intracranial venous malformation. Retin Cases Brief Rep.

[5] Thanos A, Randhawa S, Drenser KA (2016). Macular retinal cavernous hemangioma associated with congenital retinal macrovessel. JAMA Ophthalmol.

[6] Medina-Tapia A, Molina-Sócola FE, Llerena-Manzorro L (2017). Macrovaso retiniano asociado a telangiectasias periféricas retinianas e isquemia retiniana. Archivos de la Sociedad Española de Oftalmología.

[7] Petropoulos I, Petkou D, Theoulakis P, Kordelou A, Pournaras C, Katsimpris J (2008). Congenital retinal macrovessels: description of three cases and review of the literature. Klin Monatsbl Augenheilkd.

[8] Cho K, Bae K, Kim JM, Kang SW (2017). A case of retinal cavernous hemangioma diagnosed with spectral domain optical coherence tomography. Korean J Ophthalmol.

[9] Shields JA, Eagle RC, Ewing MQ (2014). Retinal cavernous hemangioma: fifty-two years of clinical follow-up with clini¬copathologic correlation. Retina.

[10] Cennamo G, Amoroso F, Solari D, Alfieri M, de Crecchio G (2017). Optical coherence tomography angiography in retinal cavernous hemangioma. Int J Ophthalmol.

[11] Li J, Li Y, Li H (2019). New interpretation of multimodality fundus imaging for retinal cavernous hemangioma. Curr Eye Res.

